# Association study to evaluate TFPI gene in CAD in Han Chinese

**DOI:** 10.1186/s12872-017-0626-y

**Published:** 2017-07-17

**Authors:** Ying Zhao, Yanbo Yu, Maowei Shi, Xi Yang, Xueqi Li, Feng Jiang, Yundai Chen, Xiaoli Tian

**Affiliations:** 1 Department of Geriatrics, Jinan Military General Hospital, Jinan, 250031 China; 2 Department of Gastroenterology, Qilu Hospital of Shandong University, Jinan, 250012 China; 3 Department of Human Population Genetics, Institute of Molecular Medicine, Peking University, Beijing, 100871 China; 4 Department of Cardiology, the Fourth Affiliated Hospital of Harbin Medical University, Harbin, 150001 China; 5 Department of Cardiology, Chinese PLA General Hospital, Beijing, 100853 China

**Keywords:** Tissue factor pathway inhibitor, Coronary artery disease, Single nucleotide polymorphism, Han Chinese

## Abstract

**Background:**

Tissue factor pathway inhibitor (TFPI) is the main physiological inhibitor of TF-induced blood coagulation process, and may play essential roles in the pathogenesis of major adverse cardiac events. This study was designed to determine whether the variation of TFPI was related with coronary artery disease (CAD) in the Han Chinese populations.

**Methods:**

A total of 1271 patients with coronary atherosclerosis and 1287 normal individuals from northern China were enrolled in the present study. Four tagging single-nucleotide polymorphisms (SNPs) (rs7586970, rs6434222, rs10153820 and rs8176528) from TFPI were selected and genotyped by direct sequencing. And the genotypes of the above SNPs were determined in all these participants.

**Results:**

In the populations from Beijing and Harbin**,** no significant case-control differences in the frequencies of TFPI polymorphism (rs10153820 and rs8176528) were observed between CAD patients and controls. Meanwhile, two SNPs of TFPI (rs7586970 and rs6434222) were found to be associated with CAD in both groups. In stratified analyses based on gender, smoking, hypertension, diabetes mellitus and hyperlipidemia, we further determined that the investigated genetic variations of the TFPI genes seemed to be related with diabetes mellitus in CAD patients.

**Conclusions:**

Genetic variations of the TFPI genes seem to be related with CAD, which likely cooperate with metabolic risk factor (diabetes mellitus) and play critical roles in the pathogenesis of coronary artery disease.

## Background

Over the past few years, coronary artery disease (CAD) has become a major public health problem and has been associated with increased mortality globally [1]. Evidence shows that atherosclerosis, a chronic inflammatory disease of the arterial vessel wall, is the main cause of CAD [2, 3]. During the atherosclerotic process, chronic inflammatory responses are often related with the development of thrombus-mediated acute coronary events. Rupture or erosion of atherosclerotic plaques or endothelial cell damage can cause exposure of subendothelial procoagulants such as tissue factor (TF) to circulating blood, followed by the activation of the coagulation process, leading to thrombin formation and subsequent acute coronary occlusion [4].

TF mediated activation of the coagulation cascade is inhibited by its endogenous physiological inhibitor, tissue factor pathway inhibitor (TFPI) [5, 6]. TFPI is constitutively synthesized by the microvascular endothelial cells. Most of the TFPI is bound to the vascular endothelium and only 20–30% of TFPI is in free forms. TFPI is a circulating, Kunitz-type protease inhibitor, acting as a natural anticoagulant that plays a major role in atherosclerotic plaques [6]. Studies showed that the administration of exogenous TFPI or of the TFPI gene could reduce the restenosis and prevent the immediate thrombus formation after balloon injury to the rabbit aortic neointima [7–9]. Meanwhile, heterozygous TFPI deficiency in atherosclerosis-prone mice exhibited a greater atherosclerotic burden, increased plaque tissue factor activity and decreased time to occlusive thrombosis after photochemical vascular injury [10, 11]. These studies indicate that TFPI attenuates TF activity and acts as a potential modulator of both atherosclerosis and arterial thrombosis.

Besides simply counteracting the role of TF, experimental data describes certain novel roles for TFPI, such as innate immunity, angiogenesis and lipid metabolism. In a cecal ligation and puncture model of peritonitis, recombinant human TFPI treated mice showed decreased plasma IL-6 levels and subsequently the mortality rate was improved [12]. Exogenous TFPI at higher physiological concentrations inhibits endothelial cell migration and tube formation in vitro, showing effects of inhibiting angiogenesis [13, 14]. Besides, TFPI could bind to lipoprotein and therefore was called lipoprotein associated coagulation inhibitor (LACI). In a murine model of flow cessation, upregulation of TFPI has been shown to reduce the development of arterial thrombosis and inhibit vascular remodeling associated with flow interruption [15]. On the contrary, TFPI deficiency demonstrated a greater atherosclerotic burden in atherosclerosis-prone Apo E (−/−) mice [11]. Furthermore, association studies demonstrated that TFPI was significantly higher with older age, male gender, increased low-density lipoprotein(LDL), current smoking and diabetes [16, 17].

The mechanism of coronary artery disease is of a complicated nature. Consistent findings indicated a role for TFPI in the pathogenesis of atherosclerosis development, not only counteracting the role of TF but also acting as an anti-inflammatory, anti-angiogenic and lipid-lowering substance. Searching for the genetic variants has been recognized as an essential strategy for the prediction, prevention and individualized treatment of CAD. Hence, in this study, we are determined to explore whether TFPI polymorphisms could influence the risk of CAD in the Han Chinese populations. We selected four tagging SNPs of TFPI (rs7586970, rs6434222, rs10153820 and rs8176528). The frequencies of TFPI were evaluated in Chinese CAD patients from two geographically isolated regions of northern China.

## Methods

### Population and the definition of risk factors

The cases in the present study were hospitalized patients who accepted X-ray coronary angiography for diagnostic purposes from two medical centers located in Beijing and Harbin. The normal controls were selected among hospital employees and blood donors with normal X-ray coronary angiography from the two medical centers. All subjects were Han Chinese coming from northern China. The inclusion and exclusion criteria of CAD and the diagnostic criteria for relevant risk factors were clearly stated in our previous study [18].

This study was registered at the website www.clinicaltrials.gov (NCT 02961127) and was approved by the clinical ethical committee of the PLA General Hospital and the ethical committee of Harbin Medical University. And all subjects gave written informed consent before participation.

### Genotyping of SNPs

Details on genotyping have previously been described [18]. Human genomic DNA was extracted from EDTA-anticoagulated blood sample on the Magna Pure LC Instrument [19]. In view of the hapmap (CHB + JPT), the four tagging single-nucleotide polymorphisms (SNPs) of TFPI (rs7586970, rs6434222, rs10153820 and rs8176528) were selected. DNA fragments of 120-180 bp containing the above SNPs were selected and amplified by PCR, with the corresponding primers listed in Table 1.

**Table 1:** The pairs of PCR primers for amplifications of SNPs for TFPI

SNP	Gene	Position	primer
rs8176528	TFPI	intron	forward: 5′- CAGTTCGTGTAGGGTTACTCAT −3’
			reverse:5′- CCAGAGACTTTATGAGTGTCT −3’
rs10153820	TFPI	5′ upstream region	forward: 5′-CGTTGGAGGTCTCTCTTAGT-3’
			reverse:5′- CTGGGCTGAGTAGCCAAGTT-3’
rs6434222	TFPI	intron	forward: 5′-GTTTGGTTCAAGAGAGGAACT-3’
			reverse:5′- CATGACTCAGCTGCCAGGACT-3’
rs7586970	TFPI	Serine to Asn	forward: 5′- GAAGGCGTTCAGAAAGACTTGGT-3’
			reverse:5′-CCCTCAGCATTGACCACAGT-3’

The amplified DNA fragments were subsequently purified by PEG precipitation and subjected to direct sequencing with a BigDye v3.1 kit and running on ABI 3130XL.

### Statistical analysis

Values are expressed as the mean ± standard deviation or otherwise stated. Univariate analysis of the general characteristics of the population involves the independent Student t test or chi-square test as applicable. Genotype distribution for single SNPs was analyzed for departure from the Hardy-Weinberg equilibrium using the chi-square test. All statistical analyses involved use of SPSS statistical package version 17.0 (SPSS Inc., Chicago, IL, UAS). The significance level was taken to be *p* < 0.05.

## Results

### Characteristics of the study population

The clinical characteristics of all the included individuals are shown in Table 2. Two pairs of CAD patients and non-CAD normal controls were recruited among the Han Chinese from the two hospitals in Beijing and Harbin. One pair (Population 1) was collected from northern China while the other (Population 2) was collected from north-eastern China. Population 1 consisted of 808 cases and 829 non-CAD controls whereas Population 2 consisted of 463 cases and 458 non-CAD controls. Both Population 1 and Population 2 were age and gender matched. The risk factors were compared between cases and normal controls by t test (age and BMI) and Chi-square test (gender, smoking, hypertension, diabetes mellitus and hyperlipidemia).

**Table 2:** Characteristics of study populations

	Population 1			Population 2		
	case (*n* = 808)	control (*n* = 829)	*P* value	case (*n* = 463)	control (*n* = 458)	*P* value
age (year)	60.36 ± 10.22	61.12 ± 12.01	0.166	54.06 ± 8.76	53.27 ± 9.06	0.175
male	634 (78.5%)	647 (78.0%)	0.837	335 (72.4%)	332 (72.5%)	0.963
BMI (kg/m^2^)	25.70 ± 3.28	24.97 ± 3.08	<0.001	25.56 ± 3.26	24.20 ± 2.89	<0.001
smoking	367 (45.4%)	111 (13.4%)	<0.001	269 (58.1%)	232 (50.7%)	<0.001
Hypertension	528 (65.3%)	311 (37.5%)	<0.001	294 (63.5%)	118 (25.8%)	<0.001
diabetes mellitus	225 (27.8%)	104 (12.5%)	<0.001	125 (27.0%)	30 (6.6%)	<0.001
hyperlipidemia	439 (54.3%)	521 (62.8%)	<0.001	314 (67.8%)	181 (39.5%)	<0.001

The data were presented as mean ± SEM (standard error of the mean) for age and BMI as well as No.(percentage) for other factors. *P* values for age and BMI were calculated from t-test comparing case and control groups within population. *P* values for gender, smoking, hypertension, diabetes mellitus, hyperlipidemia were calculated from Chi-square test within population. BMI: body mass index, which is calculated by body weight (Kg)/ height^2^ (m^2^)

### Genotype distribution and genotype association analysis

In both populations from Beijing and Harbin, no significant deviation among the four tagging SNPs of TFPI was found by the Hardy-Weinberg equilibrium test. The distribution of the TFPI genotype among patients and normal controls in both regions is demonstrated in Table 3. No statistically significant differences in the frequencies of rs8176528 and rs10153820 were obtained between CAD cases and non-CAD controls (rs8176528, *p* = 0.146 for population 1 and 0.486 for population 2; rs10153820, *p* = 0.792 for population 1 and 0.959 for population 2, Table 3), while statistically significant differences were obtained in the frequencies of rs6434222 and rs7586970 between the two populations from Beijing and Harbin (rs6434222, *p* < 0.001 for population 1 and population 2; rs7586970, *p* = 0.020 for population 1 and 0.018 for population 2, Table 3).

**Table 3:** Frequency of TFPI polymorphism in CAD population from two regions

		Population 1			Population 2		
SNP	genotype	CAD n (%)	Non-CAD n (%)	P	CAD n (%)	Non-CAD n (%)	P
rs8176528		808	829		463	458	
	GG	656 (81.2)	694 (83.7)	0.146	380 (82.1)	384 (83.8)	0.486
	AA GA	40 (5.0) 112 (13.8)	46 (5.6) 89 (10.7)		18 (3.9) 65 (14.0)	21 (4.6) 53 (11.6)	
Allelic A frequency (%)		11.8	10.9		10.9	10.3	
rs10153820		808	829		463	458	
	GG	415 (51.4)	420 (50.7)	0.792	285 (61.6)	286 (62.5)	0.959
	AA GA	48 (5.9) 345 (42.7)	56 (6.7) 353 (42.6)		81 (17.5) 97 (20.9)	79 (17.2) 93 (20.3)	
Allelic A frequency (%)		27.3	28.0		27.9	27.4	
rs6434222		808	829		463	458	
	TT	433 (53.6)	496 (59.8)	<0.001	235 (50.7)	285 (62.2)	<0.001
	AA TA	48 (5.9) 327 (40.5)	99 (11.9) 234 (28.3)		11 (2.4) 217 (46.9)	60 (13.1) 113 (24.7)	
Allelic A frequency (%)		26.2	26.1		25.8	25.4	
rs7586970		808	829		463	458	
	TT	681 (84.3)	703 (84.8)	0.020	384 (82.9)	391 (85.4)	0.018
	CC TC	36 (4.5) 91 (11.2)	57 (6.9) 69 (8.3)		21 (4.5) 58 (12.6)	32 (7.0) 35 (7.6)	
Allelic C frequency (%)		10.1	11.0		10.8	10.8	

Calculations are performed with comparison of three different genotypes. Values are the number (percentage) of subjects. Significant differences were drawn in frequencies of rs7586970 and rs6434222 between CAD cases and non-CAD controls

For better understanding the link between the investigated SNPs and other risk factors in CAD patients, we further performed stratification analyses based on gender, smoking, medical history of hypertension, hyperlipidemia and diabetes mellitus. Due to the influence of diabetes mellitus, a significant difference in the frequencies of TFPI SNPs was obtained in individuals with CAD compared to controls without CAD in our study (shown in Table 4). No obvious differences in the frequencies were obtained for any genotype based on gender (Table 5), smoking (Table 6), hypertension (Table 7), or hyperlipidemia (Table 8).

**Table 4:** Frequencies of TFPI polymorphisms in two populations according to diabetes mellitus

SNP	genotype	Population 1						Population 2					
		diabetes mellitus			Non- diabetes mellitus			diabetes mellitus			Non- diabetes mellitus		
		CAD n (%)	Non-CAD n(%)	*P*	CAD n (%)	Non-CAD n (%)	*P*	CAD n (%)	Non-CAD n (%)	*P*	CAD n (%)	Non-CAD n (%)	*P*
Rs8176528	GG	225	104	0.417	583	725	<0.001	125	30	0.726	338	428	0.003
		199	76		457	618		65	18		315	366	
		(88.4)	(73.1)		(78.4)	(85.2)		(52)	(60.0)		(93.2)	(85.5)	
	AA	9	5		31	41		9	2		9	19	
		(4.0)	(4.8)		(5.3)	(5.7)		(7.2)	(6.7)		(2.7)	(4.4)	
	GA	17	23		95	66		51	10		14	43	
		(7.6)	(22.1)		(16.3)	(9.1)		(40.8)	(33.3)		(4.1)	(10.1)	
Rs10153820	GG	225	104	0.002	583	725	0.063	125	30	0.038	338	428	0.392
		103	67		312	353		86	15		199	271	
		(45.8)	(64.4)		(53.5)	(48.7)		(68.8)	(50.0)		(58.9)	(63.3)	
	AA	27	13		21	43		19	4		62	75	
		(12.0)	(12.5)		(3.6)	(5.9)		(15.2)	(13.3)		(18.3)	(17.5)	
	GA	95	24		250	329		20	11		77	82	
		(42.2)	(23.1)		(42.9)	(45.4)		(16.0)	(36.7)		(22.8)	(19.2)	
		225	104		583	725		125	30		338	428	
Rs6434222	TT	107	53	0.803	326	443	<0.001	65	17	0.675	170	268	<0.001
		(47.6)	(51.0)		(55.9)	(61.1)		(52.0)	(56.7)		(50.3)	(62.6)	
	AA	21	8		27	91		5	2		6	58	
		(9.3)	(7.7)		(4.6)	(12.6)		(4.0)	(6.6)		(1.8)	(13.6)	
	TA	97	43		230	191		55	11		162	102	
		(43.1)	(41.3)		(39.5)	(26.3)		(44.0)	(36.7)		(47.9)	(23.8)	
		225	104		583	725		125	30		338	428	
Rs7586970	TT	161	76	0.926	520	627	0.043	67	13	0.065	317	378	0.027
		(71.6)	(73.1)		(89.2)	(86.5)		(53.6)	(43.3)		(93.8)	(88.3)	
	CC	13	5		23	52		13	8		8	24	
		(5.8)	(4.8)		(3.9)	(7.2)		(10.4)	(26.7)		(2.4)	(5.6)	
	TC	51	23		40	46		45	9		13	26	
		(22.6)	(22.1)		(6.9)	(6.3)		(36.0)	(30.0)		(3.8)	(6.1)	

Calculations were performed with comparison of three different genotypes. Values are the number (percentage) of subjects. After stratification analysis based on diabetes mellitus, significant association was found between genotype distributions and CAD in CAD patients and non-CAD controls

**Table 5:** Frequencies of TFPI polymorphisms in two populations according to genders

SNP	genotype	Population 1						Population 2					
		men			women			men			women		
		CAD n (%)	Non-CAD n (%)	*P*	CAD n (%)	Non-CAD n (%)	*P*	CAD n (%)	Non-CAD n (%)	*P*	CAD n (%)	Non-CAD n (%)	*P*
Rs8176528	GG	634	647	0.281	174	182	0.484	335	332	0.327	128	126	0.913
		512	538		144	156		273	281		107	103	
		(80.8)	(83.2)		(82.8)	(85.7)		(81.5)	(84.7)		(83.6)	(81.7)	
	AA	33	37		7	9		13	15		5	6	
		(5.2)	(5.7)		(4.0)	(5.0)		(3.9)	(4.5)		(3.9)	(4.8)	
	GA	89	72		23	17		49	36		16	17	
		(14.0)	(11.1)		(13.2)	(9.3)		(14.6)	(10.8)		(12.5)	(13.5)	
Rs10153820	GG	634	647	0.838	174	182	0.343	335	332	0.988	128	126	0.782
		306	319		109	101		204	201		81	85	
		(48.3)	(49.3)		(62.6)	(55.5)		(60.9)	(60.5)		(63.3)	(67.4)	
	AA	41	45		7	11		59	60		22	19	
		(6.4)	(7.0)		(4.0)	(6.0)		(17.6)	(18.1)		(17.2)	(15.1)	
	GA	287	283		58	70		72	71		25	22	
		(45.3)	(43.7)		(33.4)	(38.5)		(21.5)	(21.4)		(19.5)	(17.5)	
Rs6434222	TT	634	647	<0.001	174	182	0.003	335	332	<0.001	128	126	0.001
		312	379		121	117		166	214		69	71	
		(49.2)	(58.6)		(69.5)	(64.3)		(49.6)	(64.5)		(53.9)	(56.3)	
	AA	41	73		7	26		7	41		4	19	
		(6.5)	(11.3)		(4.1)	(14.3)		(2.0)	(12.3)		(3.1)	(15.1)	
	TA	281	195		46	39		162	77		55	36	
		(44.3)	(30.1)		(26.4)	(21.4)		(48.4)	(23.2)		(43.0)	(28.6)	
Rs7586970	TT	634	647	0.068	174	182	0.263	335	332	0.056	128	126	0.084
		537	549		144	154		293	289		91	102	
		(84.7)	(84.9)		(82.8)	(84.6)		(87.5)	(87.0)		(71.1)	(81.0)	
	CC	28	44		8	13		10	21		11	11	
		(4.4)	(6.8)		(4.6)	(7.2)		(3.0)	(6.4)		(8.6)	(8.7)	
	TC	69	54		22	15		32	22		26	13	
		(10.9)	(8.3)		(12.6)	(8.2)		(9.5)	(6.6)		(20.3)	(10.3)	

Calculations were performed with comparison of three different genotypes. Values are the number (percentage) of subjects. After stratification analysis based on gender, no significant association was found between genotype distributions and CAD in CAD patients and non-CAD controls

**Table 6:** Frequencies of TFPI polymorphisms in two populations according to smoking status

SNP	genotype	Population 1						Population 2					
		smoking			Non- smoking			smoking			Non- smoking		
		CAD n (%)	Non-CAD n (%)	*P*	CAD n (%)	Non-CAD n (%)	*P*	CAD n (%)	Non-CAD n (%)	*P*	CAD n (%)	Non-CAD n (%)	*P*
Rs8176528	GG	367	111	0.695	441	718	0.273	269	232	0.581	194	226	0.063
		298	93		358	601		233	194		147	190	
		(81.2)	(83.8)		(81.2)	(83.7)		(86.6)	(83.6)		(75.8)	(84.1)	
	AA	18	6		22	40		7	9		11	12	
		(4.9)	(5.4)		(5.0)	(5.6)		(2.6)	(3.9)		(5.7)	(5.3)	
	GA	51	12		61	77		29	29		36	24	
		(13.9)	(10.8)		(13.8)	(10.7)		(10.8)	(12.5)		(18.5)	(10.6)	
Rs10153820	GG	367	111	0.998	441	718	0.827	269	232	0.871	194	226	0.988
		184	56		231	364		164	146		121	140	
		(50.1)	(50.5)		(52.4)	(50.7)		(61.0)	(62.9)		(62.4)	(61.9)	
	AA	20	6		28	50		48	41		33	38	
		(5.4)	(5.4)		(6.3)	(7.0)		(17.8)	(17.7)		(17.0)	(16.9)	
	GA	163	49		182	304		57	45		40	48	
		(44.5)	(44.1)		(41.3)	(42.3)		(21.2)	(19.4)		(20.6)	(21.2)	
Rs6434222	TT	367	111	0.021	441	718	<0.001	269	232	<0.001	194	226	<0.001
		178	64		255	432		136	132		99	153	
		(48.5)	(57.7)		(57.8)	(60.2)		(50.6)	(56.9)		(51.0)	(67.7)	
	AA	20	11		28	88		7	39		4	21	
		(5.4)	(9.9)		(6.3)	(12.3)		(2.6)	(16.8)		(2.1)	(9.3)	
	TA	169	36		158	198		126	61		91	52	
		(46.1)	(32.4)		(35.9)	(27.5)		(46.8)	(26.3)		(46.9)	(23.0)	
Rs7586970	TT	367	111	0.749	441	718	0.064	269	232	0.081	194	226	0.202
		306	92		375	611		221	194		163	197	
		(83.4)	(82.9)		(85.0)	(85.1)		(82.2)	(83.7)		(84.0)	(87.2)	
	CC	17	7		19	50		12	18		9	14	
		(4.6)	(6.3)		(4.3)	(7.0)		(4.4)	(7.7)		(4.6)	(6.2)	
	TC	44	12		47	57		36	20		22	15	
		(12.0)	(10.8)		(10.7)	(7.9)		(13.4)	(8.6)		(11.4)	(6.6)	

Calculations were performed with comparison of three different genotypes. Values are the number (percentage) of subjects. After stratification analysis based on smoking status, no significant association was found between genotype distributions and CAD in CAD patients and non-CAD controls

**Table 7:** Frequencies of TFPI polymorphisms in two populations according to hypertension

SNP	genotype	Population 1						Population 2					
		hypertension			Non-hypertension			hypertension			Non-hypertension		
		CAD n (%)	Non-CAD n (%)	*P*	CAD n (%)	Non-CAD n (%)	*P*	CAD n (%)	Non-CAD n (%)	*P*	CAD n (%)	Non-CAD n (%)	*P*
Rs8176528	GG	528	311	0.074	280	518	0.403	294	118	0.477	169	340	0.707
		428	271		228	423		238	100		142	284	
		(81.1)	(87.1)		(81.4)	(81.7)		(81.0)	(84.7)		(84.0)	(83.5)	
	AA	26	10		14	36		13	6		5	15	
		(4.9)	(3.2)		(5.0)	(6.9)		(4.4)	(5.1)		(3.0)	(4.4)	
	GA	74	30		38	59		43	12		22	41	
		(14.0)	(9.7)		(13.6)	(11.4)		(14.6)	(10.2)		(13.0)	(12.1)	
Rs10153820	GG	528	311	0.327	280	518	0.381	294	118	0.744	169	340	0.797
		268	174		147	246		175	75		110	211	
		(50.8)	(55.9)		(52.5)	(47.5)		(59.5)	(63.6)		(65.1)	(62.1)	
	AA	31	18		17	38		54	19		27	60	
		(5.8)	(5.8)		(6.1)	(7.3)		(18.4)	(16.1)		(16.0)	(17.6)	
	GA	229	119		116	234		65	24		32	69	
		(43.4)	(38.3)		(41.4)	(45.2)		(22.1)	(20.3)		(18.9)	(20.3)	
Rs6434222	TT	528	311	<0.001	280	518	0.002	294	118	<0.001	169	340	<0.001
		274	194		159	302		146	60		89	225	
		(51.9)	(62.4)		(56.8)	(58.3)		(49.7)	(50.8)		(52.7)	(66.2)	
	AA	33	37		15	62		8 (2.7)	21		3	39	
		(6.3)	(11.9)		(5.4)	(12.0)			(17.8)		(1.8)	(11.5)	
	TA	221	80		106	154		140	37		77	76	
		(41.8)	(25.7)		(37.8)	(29.7)		(47.6)	(31.4)		(45.5)	(22.3)	
Rs7586970	TT	528	311	0.250	280	518	0.078	294	118	0.312	169	340	0.158
		448	262		233	441		241	98		143	293	
		(84.8)	(84.2)		(83.3)	(85.2)		(82.0)	(83.1)		(84.6)	(86.2)	
	CC	23	21		13	36		14	9		7	23	
		(4.4)	(6.8)		(4.6)	(6.9)		(4.8)	(7.6)		(4.1)	(6.8)	
	TC	57	28		34	41		39	11		19	24	
		(10.8)	(9.0)		(12.1)	(7.9)		(13.2)	(9.3)		(11.3)	(7.0)	

Calculations were performed with comparison of three different genotypes. Values are the number (percentage) of subjects. After stratification analysis based on hypertension, no significant association was found between genotype distributions and CAD in CAD patients and non-CAD controls

**Table 8:** Frequencies of TFPI polymorphisms in two populations according to hyperlipidemia

SNP	genotype	Population 1						Population 2					
		hyperlipidemia			Non- hyperlipidemia			hyperlipidemia			Non- hyperlipidemia		
		CAD n (%)	Non-CAD n (%)	*P*	CAD n (%)	Non-CAD n (%)	*P*	CAD n (%)	Non-CAD n (%)	*P*	CAD n (%)	Non-CAD n (%)	*P*
Rs8176528	GG	439	521	0.074	369	308	0.651	314	181	0.580	149	277	0.166
		356	447		300	247		269	157		111	227	
		(81.1)	(85.8)		(81.3)	(80.2)		(85.7)	(86.7)		(74.5)	(81.9)	
	AA	21	25		19	21		10	8		8	13	
		(4.8)	(4.8)		(5.1)	(6.8)		(3.2)	(4.4)		(5.4)	(4.7)	
	GA	62	49		50	40		35	16		30	37	
		(14.1)	(9.4)		(13.6)	(13.0)		(11.1)	(8.9)		(20.1)	(13.4)	
Rs10153820	GG	439	521	0.301	369	308	0.848	314	181	0.788	149	277	0.688
		217	251		198	169		196	110		89	176	
		(49.4)	(48.2)		(53.7)	(54.9)		(62.4)	(60.8)		(59.7)	(63.5)	
	AA	20	36		28	20		53	35		28	44	
		(4.6)	(6.9)		(7.6)	(6.5)		(16.9)	(19.3)		(18.8)	(15.9)	
	GA	202	234		143	119		65	36		32	57	
		(46.0)	(44.9)		(38.7)	(38.6)		(20.7)	(19.9)		(21.5)	(20.6)	
		439	521		369	308		314	181		149	277	
Rs6434222	TT	252	321	0.002	181	175	<0.001	158	125	<0.001	77	160	<0.001
		(57.4)	(61.6)		(49.1)	(56.8)		(50.3)	(69.1)		(51.7)	(57.8)	
	AA	28	58		20	41		6	19		5	41	
		(6.4)	(11.1)		(5.4)	(13.3)		(1.9)	(10.5)		(3.4)	(14.8)	
	TA	159	142		168	92		150	37		67	76	
		(36.2)	(27.3)		(45.5)	(29.9)		(47.8)	(20.4)		(44.9)	(27.4)	
Rs7586970	TT	439	521	0.117	369	308	0.125	314	181	0.064	149	277	0.073
		372	449		309	254		252	160		132	231	
		(84.7)	(86.2)		(83.7)	(82.5)		(80.3)	(88.4)		(88.6)	(83.4)	
	CC	19	32		17	25		16	6		5	26	
		(4.3)	(6.1)		(4.6)	(8.1)		(5.1)	(3.3)		(3.4)	(9.4)	
	TC	48	40		43	29		46	15		12	20	
		(11.0)	(7.7)		(11.7)	(9.4)		(14.6)	(8.3)		(8.0)	(7.2)	

Calculations were performed with comparison of three different genotypes. Values are the number (percentage) of subjects. After stratification analysis based on hyperlipidemia, no significant association was found between genotype distributions and CAD in CAD patients and non-CAD controls

## Discussion

Our present study investigated four tagging SNPs of TFPI in CAD Han Chinese patients from two medical centers in Beijing and Harbin. We demonstrated for the first time that significant differences were drawn in the frequencies of rs7586970 and rs6434222 between CAD cases and non-CAD controls from two geographically isolated regions. For better understanding the interaction between genetic variations and other risk factors, stratification analysis was further applied and significant differences in four genotype distributions were found in patients with type 2 diabetes mellitus compared with non-DM controls. These results provided the first evidence that genetic variations of the TFPI genes are associated with the risk of CAD in Han Chinese patients.

The possible interactions between the genetic variations and the onset of CAD have been increasingly studied over the past few years. These studies strongly suggest that genetic variations can contribute to the pathogenesis of CAD, thereby may act as an indicator to predict the onset of the disease. CAD is a chronic inflammatory process resulting from the interactions between lipoprotein metabolism, plaque rupture and thrombosis [20]. Due to the complicated etiology, exploring the possible genetic polymorphisms may be beneficial to understand the variant individual susceptibility to risk factors that cause CAD. In one study, whole genome scans were performed trying to identify the candidate genetic loci related with hypertension, hyperlipidemia, low HDL levels and diabetes [21]. However, up to now, few genetic loci with obvious susceptibility of CAD have been confirmed, emphasizing the diversity and complexity of the disease.

The TFPI gene comprises 9 exons separated by 8 introns with a promoter region. Mature TFPI molecule comprises three tandem Kunitz-type domains. The comprising elements of TFPI are listed as follows: a negatively charged NH2-terminal region connected by the first Kunitz-type domain (K1), a linker domain, a second Kunitz-type domain (K2), a second linker domain, the third Kunitz-type domain (K3) and a positively charged COOH-terminal basic region. As is known, the majority of TFPI is synthesized by vascular endothelial cells and smooth muscle cells [22, 23]. TFPI co-localizes with endothelial cells and macrophages in human atherosclerotic plaques, where it may modulate atherosclerosis and arterial thrombosis by attenuating TF activity [24, 25]. Several investigations focusing on the association between polymorphisms of the TFPI and cardiovascular diseases have been done to make clear the crucial role of TFPI. For instance, in Germany, the polymorphisms of P151L located in TFPI have been put forward in patients with venous thrombosis [26]. Another study carried out in France screened the TFPI gene, V264 M for point sequence variations among patients with acute coronary syndrome. Unfortunately, the result did not demonstrate that the variations of TFPI contribute to acute coronary syndromes [27]. Whether TFPI variations are associated with the susceptibility of CAD still remains unclear.

To explore the link between TFPI gene variations and coronary heart disease, the detection of 4 tagging SNPs (rs7586970, rs6434222, rs10153820 and rs8176528) was executed in this study. And we found that frequencies of rs7586970 and rs6434222 showed significant difference in Chinese CAD patients, indicating that the information of the TFPI gene polymorphism was helpful for evaluating the risk of developing coronary heart disease in Han Chinese. Previously, Jia Yu et al. investigated the link between TFPI-2 gene variations and atherosclerosis in the Chinese population, and two SNPs (rs59805398 and rs34489123) and 5 haplotypes were confirmed to be correlated with CAD. Moreover, TFPI-2 gene polymorphisms might not predict the severity of coronary atherosclerosis [28]. Trine B. Opstad et al. demonstrated a significant influence of the TFPI polymorphisms on thrombin generation, which might be an outcome of the reported genotype-induced alterations in the blood TFPI levels, suggesting a modified risk of atherothrombosis in patients holding the TFPI-399 and TFPI-33 polymorphisms [29]. Didier et al. found that the T-287C variations in the 5′ regulatory region of the TFPI gene were correlated with significant upregulation of the TFPI molecules, suggesting a positive influence of this polymorphism on the TFPI antigen expression. Though the study demonstrated that the T-287C variations were not correlated with an increased incidence of coronary artery disease, the results have not excluded the possibility that other gene variations in the TFPI may influence this incidence [30]. In the present study, the results showed for the first time that TFPI gene polymorphism (rs7586970 and rs6434222) could substantially influence the risk of atherosclerosis in Han Chinese.

Most previous studies supported that the higher levels of TFPI is associated with male gender, increased LDL, smoking and diabetes, all of which are widely accepted as cardiovascular risk factors [31]. Hence, we further investigated whether certain selected SNPs in the TFPI gene was related with cardiovascular risk factors (e.g. gender, smoking, medical history of hypertension, diabetes mellitus and hyperlipidemia) among our enrolled participants. And we found that the investigated genetic polymorphisms of the TFPI genes seemed to be related with diabetes mellitus in our enrolled CAD Han Chinese patients.

The association between CAD and diabetes mellitus has been well established. However, the detailed underlying mechanism accounting for this association has not been fully investigated. Evidence showed that patients with diabetes mellitus were related with faster aortic stenosis progression, endothelial dysfunction, higher coronary artery calcium scores and aortic valve calcification [32, 33]. The accelerated atherosclerotic process presented in patients with type 2 diabetes mellitus might be a consequence of permanent blood hyperglycemia [34]. Chronic blood hyperglycemia may result in the glycosylation of albumin, which has been confirmed to promote the production of TFPI in endothelial cells and monocytes [35]. In chronic hyperglycemia particularly in patients with microalbuminuria, the binding of advanced glycated end products could promote the infiltration of peripheral monocytes into the early atherosclerotic lesions and therefore induce an intravascular oxidative stress response, resulting in increased TFPI activity in vitro [36, 37]. Several studies reported that significantly higher TFPI plasma levels have been found in CAD patients complicated with T2DM compared to uncomplicated CAD patients [38–40]. Increased TFPI plasma levels reflect endothelial damage or impaired binding of TFPI to vascular endothelial cells by glycosaminoglycans since TFPI is predominantly produced by vascular endothelium [16, 37]. Thus, the possible expression alterations of TFPI due to genetic polymorphisms, might lead to a hypercoagulable state in CAD patients, which might be more essential for CAD patients complicated with diabetes.

In our study, the possible link between TFPI genetic polymorphism and other metabolic risk factors (e.g. gender, smoking, hypertension and hyperlipidemia) was investigated, and the results showed no evidence indicating a relationship between TFPI variations and those risk factors. However, it should be noteworthy that the participants were volunteers selected from two regions of China, and may not stand for the whole population. Hence, the association of TFPI with cardiovascular risk factors should be analyzed in more ethnic groups and in larger populations in our future studies. In addition, one recent meta-analysis demonstrated that the traditional risk factors associated with culprit plaque rupture (CPR) may vary depending on the clinical presentation of the patients. For example, hypertension was the sole clinical risk factor accounting for the ST-elevated myocardial infarction (STEMI), while advanced age, diabetes mellitus and hyperlipidemia were the candidate clinical predictors in unstable angina and non ST-elevated myocardial infarction (NSTEMI). Whether the association between TFPI polymorphism and risk factors vary depending on different clinical presentations remains unknown [41]. Further investigations are needed to make clear whether TFPI variations are related with certain subtypes of CAD.

## Conclusions

In summary, we identified two new variations located in the TFPI gene among the present population of Han Chinese CAD patients. In addition, genetic polymorphisms of the TFPI gene are likely to be related with type 2 diabetes mellitus in CAD patients. Further investigations are needed to define whether interventions that target TFPI expression and activity by SNPs might retard or reverse the progression of CAD in Han Chinese patients.

## Abbreviations

TFPI: Tissue factor pathway inhibitor
CAD: Coronary artery disease
LACI: Lipoprotein associated coagulation inhibitor
PCI: Percutaneous coronary intervention
CPR: Culprit plaque rupture.
